# Laparoscopic ileocolic resection versus infliximab treatment of distal ileitis in Crohn's disease: a randomized multicenter trial (LIR!C-trial)

**DOI:** 10.1186/1471-2482-8-15

**Published:** 2008-08-22

**Authors:** Emma J Eshuis, Willem A Bemelman, Ad A van Bodegraven, Mirjam AG Sprangers, Patrick MM Bossuyt, AW Marc van Milligen de Wit, Rogier MPH Crolla, Djuna L Cahen, Liekele E Oostenbrug, Meindert N Sosef, Annet MCJ Voorburg, Paul HP Davids, C Janneke van der Woude, Johan Lange, Rosalie C Mallant, Maarten J Boom, Rob J Lieverse, Edwin S van der Zaag, Martin HMG Houben, Juda Vecht, Robert EGJM Pierik, Theo JM van Ditzhuijsen, Hubert A Prins, Willem A Marsman, Henricus B Stockmann, Menno A Brink, Esther CJ Consten, Sjoerd DJ van der Werf, Andreas WKS Marinelli, Jeroen M Jansen, Michael F Gerhards, Clemens JM Bolwerk, Laurents PS Stassen, BW Marcel Spanier, Ernst Jan Spillenaar Bilgen, Anne-Marie van Berkel, Huib A Cense, Henk A van Heukelem, Arnold van de Laar, Warner Bruins Slot, Quirijn A Eijsbouts, Nancy AM van Ooteghem, Bart van Wagensveld, Jan MH van den Brande, Anna AW van Geloven, Karien F Bruin, John K Maring, Bas Oldenburg, Richard van Hillegersberg, Dirk J de Jong, Robert Bleichrodt, Donald L van der Peet, Pascal EP Dekkers, T Hauwy Goei, Pieter CF Stokkers

**Affiliations:** 1 Department of Gastroenterology and Hepatology, Academic Medical Center, Amsterdam, The Netherlands; 2 Department of Surgery, Academic Medical Center, Amsterdam, The Netherlands; 3 Department of Gastroenterology and Hepatology, VU Medical Center, Amsterdam, The Netherlands; 4 Department of Medical Psychology, Academic Medical Center, Amsterdam, The Netherlands; 5 Department of Clinical Epidemiology and Bio-statistics, Academic Medical Center, Amsterdam, The Netherlands; 6 Department of Gastroenterology and Hepatology, Amphia Hospital, Breda, The Netherlands; 7 Department of Surgery, Amphia Hospital, Breda, The Netherlands; 8 Department of Gastroenterology and Hepatology, Amstelland hospital, Amstelveen, The Netherlands; 9 Department of Gastroenterology and Hepatology, Atrium Medical Center, Heerlen, The Netherlands; 10 Department of Surgery, Atrium Medical Center, Heerlen, The Netherlands; 11 Department of Gastroenterology and Hepatology, Diakonessen Hospital, Utrecht, The Netherlands; 12 Department of Surgery, Diakonessen Hospital, Utrecht, The Netherlands; 13 Department of Gastroenterology and Hepatology, Erasmus Medical Center, Rotterdam, The Netherlands; 14 Department of Surgery, Erasmus Medical Center, Rotterdam, The Netherlands; 15 Department of Gastroenterology and Hepatology, Flevo Hospital, Almere, The Netherlands; 16 Department of Surgery, Flevo Hospital, Almere, The Netherlands; 17 Department of Gastroenterology and Hepatology, Gelre Hospital, Apeldoorn, The Netherlands; 18 Department of Surgery, Gelre Hospital, Apeldoorn, The Netherlands; 19 Department of Gastroenterology and Hepatology, Haga Teaching Hospital, Den Haag, The Netherlands; 20 Department of Gastroenterology and Hepatology, Isala Hospital, Zwolle, The Netherlands; 21 Department of Surgery, Isala Hospital, Zwolle, The Netherlands; 22 Department of Gastroenterology and Hepatology, Jeroen Bosch Hospital, 's-Hertogenbosch, The Netherlands; 23 Department of Surgery, Jeroen Bosch Hospital, 's-Hertogenbosch, The Netherlands; 24 Department of Gastroenterology and Hepatology, Kennemer Gasthuis, Haarlem, The Netherlands; 25 Department of Surgery, Kennemer Gasthuis, Haarlem, The Netherlands; 26 Department of Gastroenterology and Hepatology, Meander Medical Center, Amersfoort, The Netherlands; 27 Department of Surgery, Meander Medical Center, Amersfoort, The Netherlands; 28 Department of Gastroenterology and Hepatology, Medical Center Haaglanden, Den Haag, The Netherlands; 29 Department of surgery, Medical Center Haaglanden, Den Haag, The Netherlands; 30 Department of Gastroenterology and Hepatology, Onze Lieve Vrouwe Gasthuis, Amsterdam, The Netherlands; 31 Department of Surgery, Onze Lieve Vrouwe Gasthuis, Amsterdam, The Netherlands; 32 Department of Gastroenterology and Hepatology, Reinier de Graaf Gasthuis, Delft, The Netherlands; 33 Department of Surgery, Reinier de Graaf Gasthuis, Delft, The Netherlands; 34 Department of Gastroenterology and Hepatology, Rijnstate Hospital, Arnhem, The Netherlands; 35 Department of Surgery, Rijnstate Hospital, Arnhem, The Netherlands; 36 Department of Gastroenterology and Hepatology, Rode Kruis Hospital, Beverwijk, The Netherlands; 37 Department of Surgery, Rode Kruis Hospital, Beverwijk, The Netherlands; 38 Department of Gastroenterology and Hepatology, Slotervaart Hospital, Amsterdam, The Netherlands; 39 Department of Surgery, Slotervaart Hospital, Amsterdam, The Netherlands; 40 Department of Gastroenterology and Hepatology, Spaarne Hospital, Hoofddorp, The Netherlands; 41 Department of Surgery, Spaarne Hospital, Hoofddorp, The Netherlands; 42 Department of Gastroenterology and Hepatology, St Lucas Andreas Hospital, Amsterdam, The Netherlands; 43 Department of Surgery, St Lucas Andreas Hospital, Amsterdam, The Netherlands; 44 Department of Gastroenterology and Hepatology, Tergooi Hospitals, Hilversum, The Netherlands; 45 Department of Surgery, Tergooi Hospitals, Hilversum, The Netherlands; 46 Department of Gastroenterology and Hepatology, Twee Steden Hospital, Tilburg, The Netherlands; 47 Department of Surgery, Twee Steden Hospital, Tilburg, The Netherlands; 48 Department of Gastroenterology and Hepatology, University Medical Center Utrecht, Utrecht, The Netherlands; 49 Department of Surgery, University Medical Center Utrecht, Utrecht, The Netherlands; 50 Department of Gastroenterology and Hepatology, St Radboud University Medical Center, Nijmegen, The Netherlands; 51 Department of Surgery, St Radboud University Medical Center, Nijmegen, The Netherlands; 52 Department of Surgery, VU Medical Center, Amsterdam, The Netherlands; 53 Department of Gastroenterology and Hepatology, Zaans Medical Center, Zaandam, The Netherlands; 54 Department of Surgery, Zaans Medical Center, Zaandam, The Netherlands

## Abstract

**Background:**

With the availability of infliximab, nowadays recurrent Crohn's disease, defined as disease refractory to immunomodulatory agents that has been treated with steroids, is generally treated with infliximab. Infliximab is an effective but expensive treatment and once started it is unclear when therapy can be discontinued. Surgical resection has been the golden standard in recurrent Crohn's disease. Laparoscopic ileocolic resection proved to be safe and is characterized by a quick symptom reduction.

The objective of this study is to compare infliximab treatment with laparoscopic ileocolic resection in patients with recurrent Crohn's disease of the distal ileum with respect to quality of life and costs.

**Methods/design:**

The study is designed as a multicenter randomized clinical trial including patients with Crohn's disease located in the terminal ileum that require infliximab treatment following recent consensus statements on inflammatory bowel disease treatment: moderate to severe disease activity in patients that fail to respond to steroid therapy or immunomodulatory therapy. Patients will be randomized to receive either infliximab or undergo a laparoscopic ileocolic resection. Primary outcomes are quality of life and costs. Secondary outcomes are hospital stay, early and late morbidity, sick leave and surgical recurrence. In order to detect an effect size of 0.5 on the Inflammatory Bowel Disease Questionnaire at a 5% two sided significance level with a power of 80%, a sample size of 65 patients per treatment group can be calculated. An economic evaluation will be performed by assessing the marginal direct medical, non-medical and time costs and the costs per Quality Adjusted Life Year (QALY) will be calculated. For both treatment strategies a cost-utility ratio will be calculated. Patients will be included from December 2007.

**Discussion:**

The LIR!C-trial is a randomized multicenter trial that will provide evidence whether infliximab treatment or surgery is the best treatment for recurrent distal ileitis in Crohn's disease.

**Trial registration:**

Nederlands Trial Register NTR1150

## Background

Crohn's disease is an inflammatory bowel disease that affects the entire gut, but mostly the terminal ileum of the small bowel is involved. Due to the chronic inflammation the affected bowel segment is scarred and may become stenotic. Although medical treatment aims to reduce the inflammation, many patients eventually will have surgery because of obstructive complaints [1].

Primary medical treatment is still considered the preferred treatment because of the potential morbidity associated with surgery. Furthermore, medical treatment might avert surgery. Medical therapy consists of remission induction by a short course of steroids most often followed by maintenance therapy with immunomodulating agents. Recurrence of disease activity is primarily treated with steroids. Frequent disease exacerbations and steroid dependency are an indication for treatment with infliximab. Infliximab is a chimeric anti-TNF monoclonal antibody against tumor necrosis factor, an important proinflammatory cytokine in Crohn's disease. Treatment with this biological is effective in inducing and maintaining response and remission in patients with moderate to severe Crohn's disease.

Infliximab therapy once initiated is best continued at 8 weeks intervals, although interval therapy is often used to reduce costs and to avoid the risks of long-term immune suppression. Major drawbacks of medical therapy are long-term use of medication with associated impairment of quality of life, morbidity and high costs. Furthermore, infliximab treatment is an open-ended medical treatment: it is unclear for how long therapy should be continued. Interrupting the treatment is undesirable since it is associated with loss of response due to anti-infliximab antibody formation [2-4]. It remains unclear in how many patients with recurrent Crohn's disease surgery can eventually be avoided [1] Thus, patients with recurrent Crohn's disease encompass a heterogeneous group of patients some of which will respond to (long-term) medical treatment whereas in others surgery cannot be averted by medical treatment.

It is well established that an ileocolic resection is an effective and low morbidity operation resulting in a quick relieve of complaints and fast restoration of quality of life. Most frequent complications requiring reoperation are anastomotic dehiscence and intra-abdominal abscess. In several publications analyzing safety of laparoscopic ileocolic resection, the percentage of complications requiring reoperation varied from 0 to 7.6% [5]. After ileocolic resection, medication can be stopped or limited to prophylactic medication when indicated [6]. The length of loss of small bowel is generally limited and averages 20–25 cm in patients who had surgery for obstructive symptoms refractory to medical treatment. Long-term surgical recurrence occurs in 20–25% over an 8–9 years period in patients refractory to medication [7,8].

Patients are generally young and in the middle of building their socioeconomic life. Disease activity with its associated complaints and long-term therapy have a pronounced effect on quality of life characterized by sick leave and non-attendance of social activities [9,10]. Patients that have a clinical recurrence after medical treatment can be considered as patients having a more severe type of the disease. To date consensus statements offer either treatment with infliximab or surgical resection in limited disease, because no comparative studies on the two alternatives exist. It can be hypothesized that surgery may avoid long-term or ineffective medical treatment improving quality of life and reducing costs. With the implementation of the laparoscopic approach, morbidity and overall costs are further reduced, and body image and cosmesis are maintained [5,11-15]. For these reasons time has come to compare laparoscopic ileocolic resection with infliximab treatment in terms of quality of life, sick leave and costs.

## Methods/design

### Study objectives

To compare, in a prospective randomized setting, the short-term and medium-term effectiveness and costs of ileocolic resection versus infliximab therapy in patients with recurrent Crohn's disease of the distal ileum. Two research questions can be defined:

*1. *How does infliximab treatment of patients with recurrent Crohn's disease of the distal ileum compare with laparoscopic ileocolic resection in terms of quality of life, hospital stay, morbidity, sick leave and surgical (re)interventions?

*2. *What are the 12 months cumulative total costs of infliximab treatment versus laparoscopic ileocolic resection in patients with recurrent Crohn's disease of the distal ileum?

### Study design

**Figure 1:**
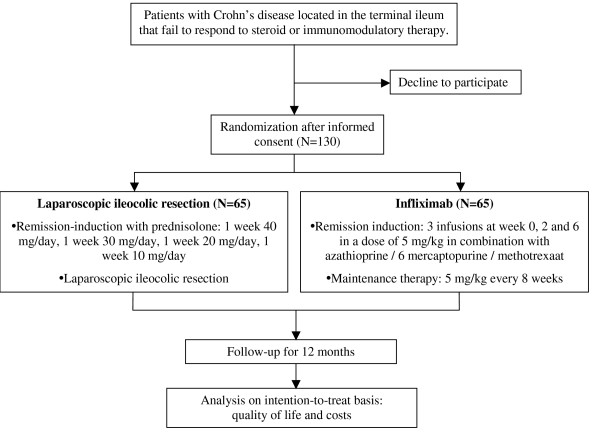
LIR!C-trial flowchart.

The LIR!C-study is a randomized multicenter trial with participation of at least five academic and 20 regional hospitals. Patients presenting with recurrent or resistant Crohn's disease of the distal ileum will be counseled and asked for informed consent if the inclusion and exclusion criteria are met. Randomization will take place after informed consent has been obtained. Patients will be randomized to either a laparoscopic ileocolic resection or to treatment with infliximab (see Figure 1).

Randomization is performed by an Internet randomization module prepared by the Department of Clinical Research and Data management. Biased-coin randomization is used and the randomization is stratified for the randomizing centers and the presence or absence of peri-anal fistulas.

### Primary and secondary endpoints

The primary endpoints of the LIR!C trial are the disease-specific quality of life as measured by the IBDQ [16,17] and the costs per QALY.

Secondary outcome parameters are general quality of life, as measured by the SF-36 [18] and EQ 5D [19,20] questionnaires; number of days on sick leave; morbidity (due to either surgery or medical treatment), total in and out hospital medical and non-medical costs and body image and cosmesis as measured by the body image questionnaire (BIQ) [13].

### Study population

The study population consists of patients with recurrent or resistant Crohn's disease of the distal ileum, not responding to immunomodulating therapy.

Inclusion criteria are: age in between 18 and 80 years, recurrent Crohn's disease of the distal ileum, a completed IBDQ, SF-36 and EQ-5D and BIQ before randomization, informed consent.

Exclusion criteria are: prior ileocolic resection for Crohn's disease, obstructive Crohn's disease of the distal ileum requiring surgery, diseased small bowel segment longer than 40 cm, abdominal abscesses and abdominal fluid collections, American Society of Anesthesiologists (ASA) III en IV, insufficient understanding of the Dutch language or cognitively inability to complete Dutch questionnaires.

### Participating centers

Twenty-seven centers will enroll patients. Five of these hospitals are academic hospitals.

### Ethics

The study is conducted in accordance with the principles of the Declaration of Helsinki and 'good clinical practice' guidelines. The protocol has been approved by the Medical Ethical Committee of the Academic Medical Center in Amsterdam and the local Ethical Committees of the participating centers. Prior to randomization informed consent will be obtained from patients.

### Study Outline

#### Recruitment

Patients will be recruited in the outpatients IBD clinics of the participating medical centers. Patients may not want to participate in the study because they reject the principle of randomization, especially since the randomization of this trial will result in two very divergent treatment strategies. A small pilot study in the AMC IBD clinic learned that most patients would participate as long as careful and clear explanation of the study is offered. In a prior study comparing laparoscopic with open ileocolic resection performed by our institute only 2 out of 62 patient refused participation or randomization [14].

#### Infliximab

Patients randomly allocated to infliximab treatment arm will undergo remission induction consisting of three subsequent infusions at week 0, 2 and 6 in a dose of 5 mg/kg. Infliximab maintenance therapy, consisting of infusions of 5 mg/kg at 8 to 12 weeks intervals, will be given to patients with active disease after an episode of disease activity that was treated with infliximab remission induction. In case of disease recurrence during infliximab treatment intervals will be shortened to 6 weeks and/or the dose level increased to 10 mg/kg. Infusion reactions will be treated with 25 mg prednisolone and 2 mg clemastine intravenously prior to subsequent infusions. Infliximab therapy will be combined with azathioprine immunomodulation in a dose of 2,5 mg/kg daily or 6-mercaptopurine 1,5 mg/kg daily. In the case of intolerance to these immunomodulating agents methotrexate will be given in an intramuscular dose of 15 mg once a week. Infliximab will be given without any co-medication in case of intolerance to abovementioned immunomodulating drugs or in case of contra-indications for the use of these drugs. Patients total blood counts and liver enzymes will be monitored 2 and 4 weeks after initiation of therapy and subsequently at 3 months intervals.

#### (Laparoscopic) ileocolic resection

Patients randomly allocated to surgery receive a short course of steroids to reduce the inflammation, consisting of prednisolone 40 mg oral dose (OD) for one week, 30 mg OD during one week, 20 mg OD for 1 week, followed by a dose of 10 mg. Once steroid therapy has been tapered to a dose of 20 mg/day ileocolic resection can be performed.

Surgery will be performed under general anesthesia. Patients will receive antibiotics for 24 hours. Ileocolic resection is done preferably laparoscopically. A variety of techniques can be applied performing a laparoscopic ileocolic resection ranging from a facilitated (laparoscopic mobilization of the right colon followed by extracorporeal vascular ligation, bowel transsection and reanastomosing) to a total laparoscopic procedure (all steps are done intracorporeally including anastomosis making). Generally, 3 or 4 trocarts suffice. The minilaparotomy preferably is done as an up and down transumbilical incision or in case of a large specimen as a Pfannenstiehl incision.

### Statistical analysis

#### Intention to treat

The analysis will be performed in accordance with the intention to treat principle.

#### Sample size calculation

The primary outcome of the study is a difference in IBDQ total score between the two randomized groups at one year. In order to assess the sample size for this study, a 0.5 between-group effect size on the IBDQ total score at week 48 was considered to be relevant. A modest effect size of 0.50 is generally considered to be clinically relevant. With a 5% two-sided significance level, 65 patients per study arm will be needed to achieve an 80% power to detect such a difference with a two-sided t-test [21]. Additional mixed-models repeated measures analysis of variance will be used to investigate whether there is a different pattern of change over time between the two study arms in the four IBDQ dimensions and the EQ 5D [22].

### Data collection and monitoring

Patients will be followed for a period of 12 months. Seven times during this follow-up period patients will complete a set of questionnaires (the IBDQ, EQ 5D, SF-36 and BIQ): patients will complete the first set of questionnaires before randomization, the next set at week 2 of their therapy, the third set at week 6 and after that every 3 months (3, 6, 9 and 12 months after start of therapy). The questionnaires will be sent to the patients by post accompanied by a return envelop provided with postage stamps and the address of the hospital. Collection of the questionnaires will be safeguarded by the trial coordinator.

Additional to the questionnaires, disease activity will be assessed by calculating the CDAI. For this calculation patients will be asked to keep a diary for seven days. In total, patients will fill in 7 diaries. During a visit to the gastro-enterologist or trial-nurse, hematocrit, presence or absence of an abdominal mass and number of complications will be assessed. CDAI can be calculated from these data combined with data from the CDAI-diaries. These visits will coincide with visits to the outpatient department for conventional patient care. At the end of the study period, after 12 months, patients will undergo an endoscopy to measure the extent of inflammation 12 months after therapy. Patients that received an ileocolic resection will be scored using the Rutgeerts endoscopic score.

Patients will be contacted by telephone every month by a trial nurse to assess complications, additional interventions, re-admissions, duration of hospital and intensive care stay and visits to the outpatient clinic, number of days of sick leave and of social in attendance and to ensure completions of the questionnaires.

An electronic Case Record Form (CRF) will include general patient's data (sex, age, medical history etc), patient's response to the questionnaires and data concerning type of intervention, complications, mortality, duration of hospital and intensive care stay.

An independent trial monitor from the IBD trial-department will monitor the study procedure and the data of included patients.

### Data analysis

As we do not expect a difference in mortality, data on quality of life will be the key outcome measure in the comparison. Differences in quality of life and morbidity will be analyzed using mixed-models analysis of variance for repeated measures, accounting for differences in survival between groups. Mortality will be compared using Kaplan-Meier curves and log-rank statistics.

To analyze the secondary outcomes (general quality of life, number of days on sick leave, morbidity (due to either surgery or medical treatment) and results of the body image questionnaire) the two groups will be compared using the statistical program SPSS 14.0^®^.

A data and safety monitoring committee will safeguard trial continuation based on safety and effectiveness data. They will perform an interim analysis after 60 included patients have reached a one month follow up.

### Economic evaluation

The marginal direct medical, non-medical and time cost, costs per QALY and cost-utility ratio will be calculated for the surgical and medical treatment strategies. Cost items will include costs of hospital admissions and readmissions (operation, nursing days, outpatient visits), institutional care (nursing homes, hospice), home care, medication and other health care providers as well as direct non-medical costs (travel expenses). Costs will be calculated by counting resource use in the diaries, questionnaires and additional 3 month interviews and multiplying these with unit prices. Standard unit prices will be used when available, complemented by results from cost calculations where needed.

The cumulative total costs will be calculated for the 12 month study period. In addition, the cumulative costs for each cost category will be calculated.

The EQ-5D score profiles will be transposed to health utility values following scoring algorithms based on time trade-off elicitation techniques applied in the general population. Both the UK and the Dutch scoring algorithms will be applied and compared in sensitivity analyses. QALY's are calculated as the product-sum of health utilities and the lengths of the preceding period in-between measurements during follow-up. In the final analysis, a 12 month difference in average QALY's will be calculated.

## Discussion

In the Netherlands infliximab treatment is indicated for patients with Crohn's disease that are either steroid refractory or steroid dependent following treatment with steroids alone or in combination with immunomodulatory drugs such as azathioprine or methotrexate [23]. These guidelines are in concert with the European consensus on infliximab treatment [4]. However, the ECCO also recommends in its consensus statement the need for trials comparing infliximab and surgery stating that 'infliximab should be considered for steroid or immunomodulatory disease or intolerance, although surgical options should be considered and discussed.' The LIR!C-trial aims to diminish this discussion and to provide an evidence-based best treatment strategy.

Besides infliximab (Remicade^®^), new biologic agents have been introduced in the past few years. Adalimumab (Humira^®^), a human anti-TNF monoclonal antibody, has possible advantages over infliximab in administration route and costs. In the future, therefore, adalimumab might become the preferred biological treatment. Nevertheless, at this moment adalimumab as therapy for Crohn's disease is relatively young and still proving itself. Four placebo-controlled trials assessing efficacy in Crohn's disease have been conducted so far, analyzing 1400 patients. Conclusions were that adalimumab is more effective than placebo for remission-induction and maintaining remission [24-27]. No trials comparing adalimumab and infliximab head to head have been published yet. Because of lack of sufficient long-term data we chose to not use adalimumab in this study. Certoluzimab Pegol (Cimzia^®^), a polyethylene glycolated anti-TNFα antibody fragment was associated with a modest improvement in response rates but with no significant improvements in remission rates if compared to placebo [28]. As maintenance therapy outcomes on response and remission were better compared to placebo-therapy [29]. However, it has not been registered in Europe yet for the therapy of Crohn's disease and therefore it is not included in this trial either.

This trial compares medical therapy with (minimal invasive) surgery for Crohn's disease of the distal ileum. The first analysis will provide short- and medium-term results up to one year of follow-up. However, since long-term data of this cohort are especially of importance, we aim to develop a follow-up study to continue follow-up after the first year.

Considering the drawbacks of infliximab treatment, ileocolic resection can be an equivalent alternative treatment, in spite of a small risk on serious surgical complications. Both strategies have not been compared in a clinical trial so far [4]. Infliximab treatment may be less cost-effective when compared to laparoscopic ileocolic resection and may show less effective when assessed by means of quality of life. Therefore this study aims to answer the question which treatment is to be preferred for recurrent distal ileitis: medical therapy or early surgery.

## Competing interests

The authors declare that they have no competing interests.

## Authors' contributions

EJE drafted the manuscript. WAB and PCFS co-authored the writing of the manuscript. PCFS and WAB are the principal investigators. All other authors participated in the design of the study during several meetings and are local investigators at the participating centers. All authors edited the manuscript and read and approved the final manuscript.

## Acknowledgements

ZonMw, grant number 10788.2201

All authors are member of the LIR!C-study group.

## Pre-publication history

The pre-publication history for this paper can be accessed here:


